# Effectiveness of Medical Music Therapy Practice: Integrative Research Using the Electronic Health Record: Rationale, Design, and Population Characteristics

**DOI:** 10.1089/jicm.2022.0701

**Published:** 2024-01-12

**Authors:** Samuel N. Rodgers-Melnick, Rachael L. Rivard, Seneca Block, Jeffery A. Dusek

**Affiliations:** ^1^ Connor Whole Health, University Hospitals of Cleveland, Cleveland, OH, USA.; ^2^ Department of Population and Quantitative Health Sciences, School of Medicine, Case Western Reserve University, Cleveland, OH, USA.; ^3^ Center for Survey and Evaluation Research, HealthPartners Institute, Minneapolis, MN, USA.; ^4^ Department of Psychiatry and School of Medicine, Case Western Reserve University, Cleveland, OH, USA.; ^5^ Department of Psychiatry Family Medicine and Community Health, School of Medicine, Case Western Reserve University, Cleveland, OH, USA.; Portions of the content of this manuscript were presented at the 2021 American Music Therapy Association National Conference on October 16, 2021.

**Keywords:** music therapy, clinical effectiveness, electronic health record

## Abstract

**Background:**

Several clinical trials support the efficacy of music therapy (MT) for improving outcomes in hospitalized patients, but few studies have evaluated the real-world delivery and integration of MT across multiple medical centers. This article describes the rationale, design, and population characteristics of a retrospective study examining the delivery and integration of MT within a large health system.

**Methods:**

A retrospective electronic health record (EHR) review was conducted of hospitalized patients seen by and/or referred to MT between January 2017 and July 2020. MT was provided across ten medical centers, including an academic medical center, a freestanding cancer center, and eight community hospitals. Discrete demographic, clinical, and MT treatment and referral characteristics were extracted from the EHR, cleaned, and organized using regular expressions functions, and they were summarized using descriptive statistics.

**Results:**

The MT team (average 11.6 clinical fulltime equivalent staff/year) provided 14,261 sessions to 7378 patients across 9091 hospitalizations. Patients were predominantly female (63.7%), White (54.3%) or Black/African American (44.0%), 63.7 ± 18.5 years of age at admission, and insured under Medicare (51.1%), Medicaid (18.1%), or private insurance (14.2%). Patients' hospitalizations (median length of stay: 5 days) were primarily for cardiovascular (11.8%), respiratory (9.9%), or musculoskeletal (8.9%) conditions. Overall, 39.4% of patients' hospital admissions included a mental health diagnosis, and 15.4% were referred to palliative care. Patients were referred by physicians (34.7%), nurses (29.4%), or advanced practice providers (24.7%) for coping (32.0%), anxiety reduction (20.4%), or pain management (10.1%). Therapists provided sessions to patients discharged from medical/surgical (74.5%), oncology (18.4%), or intensive care (5.8%) units.

**Conclusions:**

This retrospective study indicates that MT can be integrated across a large health system for addressing the needs of socioeconomically diverse patients. However, future research is needed to assess MT's impact on health care utilization (i.e., length of stay and rates of readmission) and immediate patient-reported outcomes.

## Introduction

Music therapy (MT) research has progressed significantly from early case studies demonstrating its benefits to several clinical trials establishing its efficacy for addressing patients' needs. Many randomized controlled trials (RCTs) have exhibited MT's efficacy for improving symptom management in populations including cancer;^1^ sickle cell disease;^2^ palliative care;^3^ orthopedic,^4,5^ breast,^6^ or spinal surgery;^7^ and cardiovascular care.^8,9^

Though prior studies of inpatient integrative medicine (IM) have described program development,^10^ referral patterns,^11^ delivery and integration of services,^12–14^ and effectiveness within specific populations such as cardiology^13^ and oncology,^12^ few have described the delivery and integration of inpatient MT specifically. Prior textbooks have provided recommendations for building medical MT programs.^15,16^

One quality improvement project among 57 patients receiving palliative care found that most MT referrals were for symptom management (46%), anxiety (33%), grief (7%), or psychosocial support (7%) and that clinicians perceived MT as a valuable holistic service.^17^ Another study among 106 patients receiving palliative care found that patients were most often referred for pain (28%), anxiety (18%), and relaxation (13%).^18^

However, to our knowledge, no prior investigations have described the full delivery and integration of a medical MT program provided across multiple medical centers to thousands of inpatients with a range of medical conditions using data routinely entered in the electronic health record (EHR).

We are currently conducting a large research project titled *Effectiveness of Medical Music therapy Practice: Integrative Research using the Electronic health record* (*EMMPIRE*). The first aim of EMMPIRE is a retrospective study examining the delivery and integration of MT across a large health system, and the rationale, design, and population characteristics are described in this report.

## Materials and Methods

### Setting and eligibility criteria

University Hospitals (UH) is a non-for-profit health system in Northeast Ohio serving the needs of more than 1.2 million unique patients annually. The University Hospitals Connor Whole Health (UHCWH) MT program includes 12 board-certified music therapists deployed across ten medical centers in the UH Health System. This program has been embedded within the clinical care team infrastructure as a non-pharmacologic resource for symptom management.

The MT services are provided at no cost to patients and funded through a combination of philanthropy, foundation grants, and hospitals' operating budgets. In addition, this inpatient MT program has been utilized to offer education (e.g., verbal and written descriptions of services) on available outpatient UHCWH IM modalities, including chiropractic care, massage therapy, acupuncture, and IM consults.

At the time of this study, MT was provided across ten UH medical centers, including an academic medical center, a freestanding cancer center, and eight community hospitals. Twenty-nine individuals (79.3% female) including 16 board-certified music therapists (MT-BCs) and 13 MT interns with a range of clinical experience (i.e., <1 year to over 35 years) provided MT services at various points during the 3.5-year study.

The present retrospective analysis includes all hospitalized adults (ages 18 and older) seen by and/or referred to MT within inpatient medical/surgical, oncology, or intensive care units (ICUs) between January 1, 2017 and July 30, 2020. Patients seen by and/or referred to MT solely within inpatient psychiatric, emergency department, pediatric, outpatient surgery, or outpatient clinic settings were excluded from this analysis.

Though UHCWH provided several group MT programs within inpatient psychiatric units at multiple medical centers during the study, the documentation for those sessions differed from the primary EHR documentation template used in this study (Supplementary Material) and was unavailable for extraction.

### Ethical approval

This study was approved by the UH Cleveland Medical Center Institutional Review Board as a retrospective chart review with a waiver of informed consent. This study was conducted in accordance with the Declaration of Helsinki and all relevant national regulations.

### Data collected

We extracted the following data from all records meeting eligibility criteria: (1) demographics, including age, sex, race, ethnicity, marital status, and insurance type; (2) clinical characteristics, including International Classification of Diseases Tenth Revision (ICD-10) codes for all hospital admission-related diagnoses, discharge location, length of stay, and presence of a palliative care referral; (3) MT referral data (i.e., credentials of referring providers and referral reason); and (4) MT session data, including conflict(s) of service (i.e., an attempt was made to see a patient but a session did not occur due to the patient being away from their room, asleep, busy, etc.) and use of MT interventions and/or MT education (MT Ed). Details regarding data collection, extraction, cleaning, and combination procedures are provided in Supplementary Material.

### Data analysis

Two distinct groups emerged in this retrospective study: (1) hospital admissions where MT was referred and MT was provided; and (2) hospital admissions where MT was referred, but MT was not provided. *MT referred and MT provided* was defined as: (1) documented MT referral plus documented session or (2) no documented MT referral (i.e., verbal referral) plus documented session.

*MT referred and MT not provided* was defined as: (1) documented MT referral without a documented session; (2) documented MT referral plus all MT documents were conflicts of service; or (3) no documented MT referral and all MT documents were conflicts of service. After the two groups above were defined, hospital admissions in the *MT referred and MT provided* group were subdivided further into (1) received MT Ed only and (2) received at least one MT intervention session during the hospital admission.

To summarize patients' primary diagnoses, ICD-10 codes were categorized into Major Expanded Diagnosis Clusters (MEDCs). Hospital admission records that contained an ICD-10 code for a mental health or substance use disorder as an encounter diagnosis^19^ were coded as having a mental health diagnosis. For this analysis of population characteristics among patients seen by MT, we used SAS software, Version 9.4 of the SAS System for Windows (Cary, NC) to generate descriptive statistics to summarize patients' demographics, clinical characteristics, discharge locations, referring providers, and reasons for referral.

To facilitate a comparison of our study population with hospitalization trends reported by UH in county community health needs assessments (CHNA), we separated the population into patients seen within Cuyahoga County hospitals (six mostly urban hospitals, including the academic medical center in Cleveland) and patients seen in counties outside Cuyahoga County (four hospitals serving mostly rural areas).

## Results

**FIG. 1.**
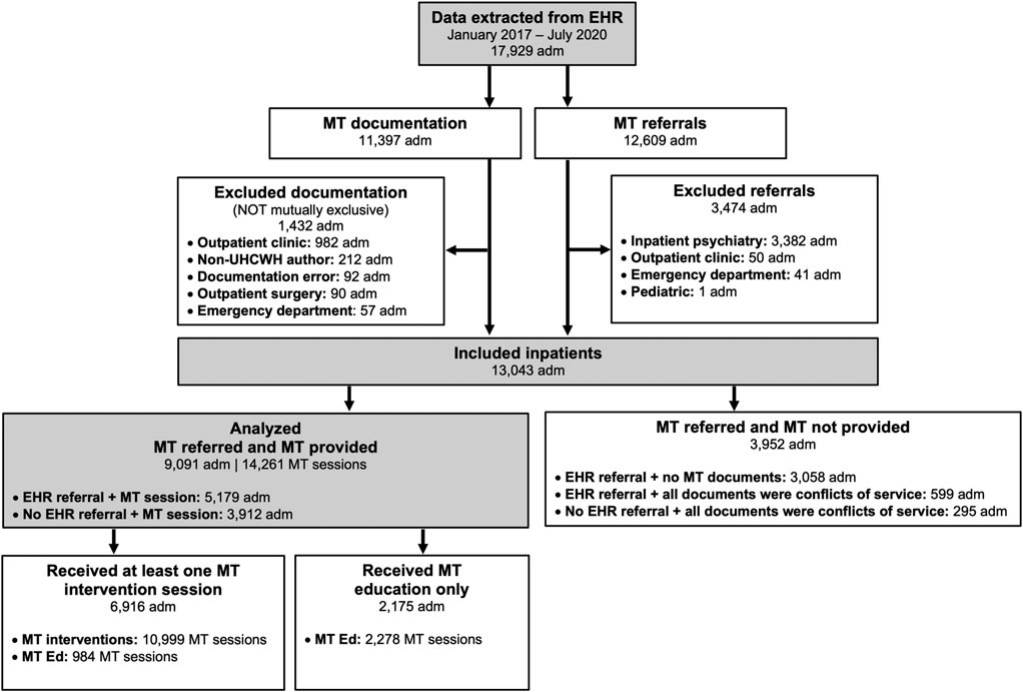
Flow diagram of study participants. adm, hospital admission; EHR, electronic health record; MT, music therapy; MT Ed, music therapy education; UHCWH, University Hospitals Connor Whole Health.

Figure 1 provides a flow chart of the hospital admissions (“adm”), referrals, and MT sessions included in this analysis. We extracted data on 17,929 hospital admissions among 13,557 unique patients. After exclusions, data on 13,043 hospital admissions among 10,337 patients were available for analysis. Of these hospital admissions, 9091 (69.7%) featured an MT session and 3952 (30.3%) did not. Of the 3952 hospital admissions that did not include an MT session, 3058 (77.4%) were instances of patients being referred to MT via the EHR and not having any MT documentation in their records.

Specific data as to why no attempt was made to provide MT to these patients were unavailable, but they likely include instances where (1) MT was referred on a unit where MT programming was not funded at the time; (2) patients were referred over the weekend or another time when therapists were unavailable; or (3) patients were referred at a time when demand for services exceeded therapists' capacity.

The remaining 894 (22.6%) hospital admissions were instances where all MT documents were considered conflicts of service. Specific conflict of service reasons within MT documentation included patients declining MT services (38.3%), being off the unit when therapists attempted to see them (18.6%), sleeping (15.1%), and receiving a procedure/working with other medical personnel in the room (5.6%).

Of the 9091 hospital admissions in which patients received MT, 6916 (76.1%) featured at least 1 MT intervention. The remaining 2175 (23.9%) featured “MT Ed” only. Though specific data indicating why these patients only received MT Ed were unavailable, this was likely due to patients being discharged or having conflicts of service following the first MT Ed session.

Thus, during the retrospective study period, the MT team (average 11.6 clinical fulltime equivalent staff/year) provided 14,261 MT sessions (10,999 MT interventions and 3262 MT Ed sessions) to 7378 patients across 9091 hospital admissions.

### Demographics

Table 1 summarizes the demographics of hospital admissions in which a patient received MT. Patients were predominantly female (63.7%), White (54.3%) or Black/African American (44.0%), 63.7 ± 18.5 years of age on average at the time of hospital admission, and insured under Medicare (51.1%), Medicaid (18.1%), or private insurance (14.2%). The proportion of patients seen by MT who identified as Black/African American within Cuyahoga County was 48.0%, whereas the proportion outside Cuyahoga County was 8.3%.

**Table 1.** Demographic Characteristics of Patients Seen by Music Therapy

Variables	All admissions (*n* = 9091)	Cuyahoga County admissions (*n* = 8172)	Outside Cuyahoga County admissions (*n* = 919)
Age (years)			
Mean ± SD	63.72 ± 18.50	63.38 ± 18.67	66.75 ± 16.63
Median [range]	66 [18–105]	65 [18–105]	68 [18–101]
18–34, *n* (%)	842 (9.3)	807 (9.9)	35 (3.8)
35–49, *n* (%)	1101 (12.1)	986 (12.1)	115 (12.5)
50–64, *n* (%)	2391 (26.3)	2155 (26.4)	236 (25.7)
65+, *n* (%)	4757 (52.3)	4224 (51.7)	533 (58.0)
Sex, *n* (%)			
Female	5790 (63.7)	5183 (63.4)	607 (66.1)
Male	3301 (36.3)	2989 (36.6)	312 (33.9)
Race,^a^ *n* (%)			
White	4933 (54.3)	4107 (50.3)	826 (89.9)
Black/African American	3999 (44.0)	3923 (48.0)	76 (8.3)
Other race	70 (0.8)	65 (0.8)	5 (0.5)
Declined/missing/unknown	42 (0.5)	37 (0.5)	5 (0.5)
Asian	25 (0.3)	25 (0.3)	0 (0.0)
American Indian/Alaska Native	14 (0.2)	12 (0.1)	2 (0.2)
Multi-racial	8 (0.1)	3 (0.0)	5 (0.5)
Ethnicity, *n* (%)			
Non-Hispanic	8839 (97.2)	7966 (97.5)	873 (95.0)
Declined/missing	135 (1.5)	115 (1.4)	20 (2.2)
Hispanic or Latino	117 (1.3)	91 (1.1)	26 (2.8)
Marital status, *n* (%)			
Single	3217 (35.4)	3019 (36.9)	198 (21.5)
Married/Life partner	2985 (32.8)	2687 (32.9)	298 (32.4)
Widowed	1609 (17.7)	1359 (16.6)	250 (27.2)
Divorced	1070 (11.8)	919 (11.2)	151 (16.4)
Separated	156 (1.7)	139 (1.7)	17 (1.8)
Unknown	54 (0.6)	49 (0.6)	5 (0.5)
Primary insurance, *n* (%)			
Medicare	4649 (51.1)	4035 (49.4)	614 (66.8)
Medicaid	1648 (18.1)	1500 (18.4)	148 (16.1)
Private	1288 (14.2)	1172 (14.3)	116 (12.6)
Missing^b^	1235 (13.6)	1231 (15.1)	4 (0.4)
Other	228 (2.5)	196 (2.4)	32 (3.5)
Self-Pay	43 (0.5)	38 (0.5)	5 (0.5)

^a^ Race, including multi-racial, is reported exactly as it was entered into the EHR.

^b^ Insurance information was not available for all hospital admissions in the retrospective analysis at the time the data were extracted from the EHR. Missing insurance information does not indicate that the patients were uninsured.

EHR, electronic health record; SD, standard deviation.

### Clinical characteristics

Table 2 summarizes the clinical characteristics of hospital admissions in which a patient received MT. Patients' hospital admissions (median length of stay 5 days) were primarily for cardiovascular (11.8%), respiratory (9.9%), musculoskeletal (8.9%), or infectious disease (8.2%) conditions. Overall, 3582 (39.4%) hospital admissions included a mental health diagnosis, and 1402 (15.4%) were referred to palliative care.

**Table 2.** Clinical Characteristics of Patients Seen by Music Therapy

Variables	All admissions (*n* = 9091)	Cuyahoga County admissions (*n* = 8172)	Outside Cuyahoga County admissions (*n* = 919)
Length of stay			
Mean ± SD	8.98 ± 11.31	9.25 ± 11.75	6.55 ± 5.56
Median [range]^a^	5 [0–156]	5 [0–156]	5 [0–41]
Primary diagnosis (MEDC), *n* (%)			
Cardiovascular	1076 (11.8)	975 (11.9)	101 (11.0)
Respiratory	902 (9.9)	781 (9.6)	121 (13.2)
Musculoskeletal	813 (8.9)	688 (8.4)	125 (13.6)
Infectious disease	748 (8.2)	660 (8.1)	88 (9.6)
Malignancies	732 (8.1)	717 (8.8)	15 (1.6)
Neurologic	714 (7.9)	663 (8.1)	51 (5.5)
General surgery	650 (7.1)	559 (6.8)	91 (9.9)
Hematologic	615 (6.8)	588 (7.2)	27 (2.9)
Gastrointestinal/hepatic	602 (6.6)	530 (6.5)	72 (7.8)
Renal	401 (4.4)	352 (4.3)	49 (5.3)
Toxic effects and adverse events	371 (4.1)	334 (4.1)	37 (4.0)
General signs and symptoms	329 (3.6)	305 (3.7)	24 (2.6)
Genito-urinary	255 (2.8)	214 (2.6)	41 (4.5)
Endocrine	249 (2.7)	224 (2.7)	25 (2.7)
Administrative	197 (2.2)	179 (2.2)	18 (2.0)
Psychosocial/mental health	95 (1.0)	87 (1.1)	8 (0.9)
Allergy	82 (0.9)	77 (0.9)	5 (0.5)
Female reproductive	56 (0.6)	52 (0.6)	4 (0.4)
Rheumatologic	50 (0.5)	46 (0.6)	4 (0.4)
Skin	43 (0.5)	39 (0.5)	4 (0.4)
Reconstructive	32 (0.4)	31 (0.4)	1 (0.1)
Ear, nose, and throat	26 (0.3)	21 (0.3)	5 (0.5)
Genetic	23 (0.3)	23 (0.3)	0 (0.0)
Nutrition	13 (0.1)	11 (0.1)	2 (0.2)
Dental	11 (0.1)	10 (0.1)	1 (0.1)
Eye	6 (0.1)	6 (0.1)	0 (0.0)
Mental health diagnosis,^b^ *n* (%)	3582 (39.4)	3145 (38.5)	437 (47.6)
Referred to palliative care, *n* (%)	1402 (15.4)	1318 (16.1)	84 (9.1)

^a^ Lengths of stay = 0 are observation admissions <24 h.

^b^ Includes principal, admitting, and/or secondary diagnoses.

MEDC, Major Expanded Diagnosis Cluster.

### Hospital locations

**FIG. 2.**
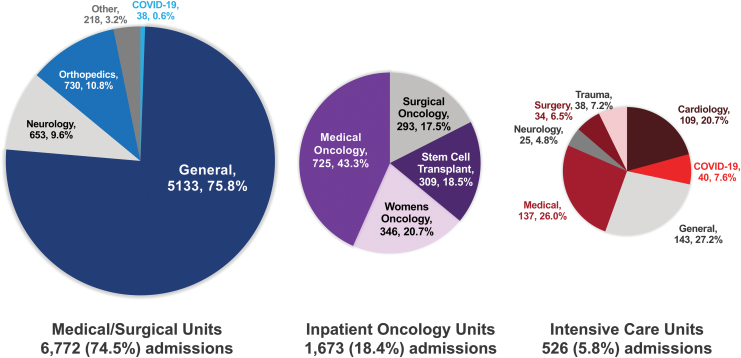
Discharge locations of patients seen by MT. Patients were seen between January 2017 and July 2020. Of all hospital admissions in which patients were seen by MT (*N* = 9091), patients were primarily discharged from medical/surgical (74.5%), inpatient oncology (18.4%), and intensive care (5.8%) units. One hundred twenty (1.3%) patients were discharged from other types of units (e.g., skilled nursing facility and inpatient rehabilitation units). Subspecialties of these types of units are indicated in the pie charts. The type and specialty of the units where patients were seen may not be indicative of patients' actual diagnosis. Patients may have been seen in multiple locations different from their discharge locations during their hospital admissions.

Figure 2 summarizes the discharge locations of patients seen by MT. Of 9091 hospital admissions, 3037 (33.4%) occurred within the academic medical center, and 6054 (66.6%) occurred within community hospitals. Music therapists provided services on 77 unique hospital units to patients discharged from medical/surgical (74.5%) units, oncology (18.4%) units, or ICUs (5.8%).

Common specialties within unit categories included orthopedics (10.8%) and neurology (9.6%) within medical/surgical units; medical oncology (43.3%), women's oncology (20.7%), stem cell transplant (18.5%), and surgical oncology (17.5%) within inpatient oncology units; and medical (26.0%), cardiology (20.7%), COVID-19 (7.6%), trauma (7.2%), surgery (6.5%), and neurology (4.8%) within ICUs.

### MT referrals

Table 3 summarizes the EHR referrals of patients seen by MT. Overall, 1169 unique health care professionals entered MT referrals. Of the 5188 EHR referrals on unique hospital admissions, the most common groups of referring health care professionals were physicians (34.7%) followed by nurses (29.4%) and advanced practice providers (24.7%). The most common reasons for referral were for coping (32.0%), anxiety reduction (20.4%), pain management (10.1%), and mood modification (8.4%).

**Table 3.** Documented Referrals Among Patients Seen by Music Therapy

Variables	All admissions where MT was electronically referred (*n* = 5188)
Number of requesting providers	1169
Requesting provider type, *n* (%)	
Physician	1800 (34.7)
Nurse	1525 (29.4)
Advanced practice provider^a^	1280 (24.7)
Other	247 (4.8)
PT, OT, or SLP	201 (3.9)
Floor leader^b^	135 (2.6)
Reason for referral, *n* (%)^c^	
Coping	1660 (32.0)
Anxiety reduction	1057 (20.4)
Pain management	522 (10.1)
Mood modification	437 (8.4)
Normalization	352 (6.8)
Self-expression	328 (6.3)
Locus of control	223 (4.3)
Spiritual support	137 (2.6)
Family support	115 (2.2)
End of life support	100 (1.9)
Other	88 (1.7)
Cognitive goals	78 (1.5)
Speech production	57 (1.1)
Procedural support	39 (0.8)
Life review	32 (0.6)
Motor skills	11 (0.2)

^a^ Includes advanced practice registered nurses and physician assistants.

^b^ Includes nurse managers and care coordinators.

^c^ Patients could be referred for more than one reason.

MT, music therapy; OT, occupational therapy; PT, physical therapy; SLP, speech language pathology.

## Discussion

The purpose of this article was to describe the rationale, design, and population characteristics of the EMMPIRE retrospective study. To our knowledge, this study includes the largest number of MT sessions provided in a hospital setting and is the first to describe the entire scope of a medical MT program. Our population characteristics demonstrate the large scale of the UHCWH MT program: 14,261 MT sessions provided to 7378 patients who were referred by 1169 health care professionals during 9091 hospital admissions across 77 hospital units within ten medical centers spanning five counties over 3.5 years. Our results describe how MT can be integrated across a range of medical settings, from medical/surgical units to more restrictive environments such as the stem cell transplant unit and COVID-19 ICUs.

Several demographic and clinical comparisons are worth noting between this population, the overall population of patients hospitalized within the five counties, and previous large (*n* > 1000) observational studies of IM delivered throughout health systems. Most patients seen by MT in this study were female (63.7%). This is consistent with reports of majority female populations being referred to^10,11^ or seen by seen by IM^12–14^ at Abbott Northwestern Hospital (ANW), an urban hospital in Minneapolis, MN.

Previous studies of inpatient IM have also shown that female patients have higher odds of receiving^13^ and being referred to IM services.^10^ The mean age of patients seen by MT (63.7 years) is similar to the mean age of 6580 patients with cardiovascular illness seen by IM at ANW (63.5 years).^13^

Black/African American representation among our patient population is significant given the historical lack of such representation within the MT literature,^20,21^ findings from a recent study in which Black patients with cancer receiving MT reported higher pre-session pain intensity than White patients,^22^ and the high priority given to addressing structural racism within the 2019 Cuyahoga County CHNA.^23^

Recent studies support the preliminary efficacy of vocal MT for improving pain-related self-efficacy, ability to participate in social activities, and pain interference among urban, predominantly Black/African American adults with chronic pain.^24,25^ Through incorporating patients' music preferences and allowing an outlet for self-expression, MT may be a more culturally relevant and accessible form of addressing the needs of hospitalized Black/African American patients. More research is needed to compare the experiences of these patients within different IM modalities.

The racial demographics of our patients reflect the communities in which MT was provided. Specifically, the proportion of patients seen by MT who identified as Black/African American within Cuyahoga County (48.0%) is reflective of the demographics of that county, where 48.3% of Cleveland (the largest city within Cuyahoga County) residents identify as Black/African American.^26^

Similarly, the proportion of patients seen by MT outside Cuyahoga County (8.3%) is reflective of the demographics of the counties outside Cuyahoga where <10% of residents identify as Black/African American.^26^ These demographic trends may help to explain why, when compared with other inpatient IM programs, UHCWH music therapists saw a greater proportion of Black/African American patients (44.0%) compared with inpatients seen at ANW (<10%),^27^ adults referred to IM at ANW (6.1%),^11^ and adults receiving nurse delivered aromatherapy across 10 hospitals in the Allina Health System (4.6%).^14^

Recent RCTs and systematic reviews support the efficacy of MT among patients hospitalized for the common MEDCs reported in our population, including patients with hematologic/oncologic,^1,28,29^ cardiovascular,^8,9,30^ respiratory,^31^ or musculoskeletal conditions,^4,5,7^ and patients receiving palliative care.^3,32^ The number of MEDCs and their respective proportions within our population demonstrate the thorough integration of MT across UH.

The highest proportion of patients in our study were hospitalized with cardiovascular illnesses (11.8%). This result is consistent with cardiovascular illness being reported as the number one principal diagnosis among all UH hospital admissions in Cuyahoga County (17.6%)^23^ and all adult hospital admissions in counties surrounding Cuyahoga (18.5%).^33–36^

MT's integration within a wide range of populations and clinical settings is significant given the reported challenges delivering integrative therapies throughout a large hospital system (e.g., complexity of the medical environment, high demand, cultural differences between conventional and complementary approaches, and difficulty sustaining financial support).^37^

A high proportion (39.4%) of hospital admissions in our study included patients with a confirmed mental health diagnosis. This rate is significantly higher than the average of 27.8% reported among all 2016 inpatient stays in the United States.^19^ Given the above average lengths of stay and costs associated with hospitalizations with coexisting substance abuse/mental health conditions,^19^ future studies should examine whether providing MT to these patients impacts length of stay/cost and whether the presence of a mental health condition predicts referral to MT.

Among the documented referrals for MT services, physicians provided the most referrals (34.7%) relative to other disciplines. However, most referrals (65.3%) came from non-physicians, including nurses (29.4%) and advanced practice providers (24.7%). During the retrospective study, music therapists provided periodic education to medical staff regarding the role of MT and appropriate reasons for MT referral, with most of these efforts being targeted toward physicians, nurses, and advanced practice providers rather than rehabilitative therapists (e.g., physical therapy [PT], occupational therapy [OT], or speech language pathology [SLP]).

In addition, given the relatively higher frequency of contact that nurses and advanced practice providers have with patients, they are uniquely positioned to identify the acute patient/family care needs and refer patients for MT services.^38^ A prior retrospective analysis of over 14,000 EHR IM referrals demonstrated a similar high proportion of nurse-initiated referrals and found that health care professionals were more likely to refer patients who displayed strong symptoms and/or did not respond to conventional treatment.^11^

Similarly, while the most common documented reason for referral in our study was coping (32.0%), a possible consequence of it being the first option in the EHR MT referral menu, symptom management needs for anxiety reduction (20.4%) and pain management (10.1%) were the second and third most prevalent respectively.

This study supports the use of the EHR as a clinical effectiveness research tool. While one prior examination of MT's effectiveness among inpatients with cancer utilized the EHR as a means of collecting patient descriptors and MT session characteristics,^39^ to our knowledge this is the first MT study to use the EHR exclusively as a data collection tool.

There are several advantages to this approach, including the minimization of documentation burden and the optimization of routine data collection for the medical team and hospital administrators. However, specific training and guidelines are required to ensure that data are consistently collected and documented. These guidelines and trainings will be addressed in Aim 2 of EMMPIRE.

Further, this study's methods (i.e., structured documentation templates and regular expressions for data mining) are generalizable to other IM modalities and health care institutions and could enable robust multi-site observational studies. However, this research will also require standardization of data collection practices (e.g., consistent patient-reported outcomes [PROs]) across institutions.

Strengths of this study include the large sample size, diversity of sociodemographic and clinical populations, novel approach to using EHR data, and lack of constraints typically imposed by RCTs. Limitations include inability to track receipt of verbal referrals, potential errors in EHR demographic information (i.e., race, ethnicity, marital status), lack of specific data on where each MT session occurred, and lack of documentation on MT services delivered on inpatient psychiatric units and in hospitals that used a previous EHR during part of the study.

Lack of demographic clarity is a challenge inherent to many EHR systems.^40^ While we are unable to correct errors in demographic data entry by other health care professionals, we will be able to capture all referrals, specific session location data, and sessions on inpatient psychiatric units across all UH medical centers in our prospective study. Further, we recognize that utilizing the EHR in this way requires a significant investment of time and expertise in data extraction, mining, and management. The skills, resources, and EHR infrastructure needed to conduct this research may not be available within other health systems.

## Conclusions

This retrospective study supports MT's ability to be integrated across a large health system for addressing the needs of socioeconomically diverse patients. Future research is needed to (1) assess the effectiveness of various MT interventions on PROs while accounting for covariates including pain medications, demographics, clinical characteristics, and social determinants of health; (2) examine the possible effects on PROs, length of stay, and cost within specific clinical populations; and (3) optimize MT's implementation throughout patients' hospitalizations.

## Data Availability

The datasets generated and/or analyzed during the current study are not publicly available due to privacy restrictions, as the databases contain information that could compromise the privacy of research participants. However, the de-identified datasets are available from the corresponding author on reasonable request.

## Supplementary Material

Supplemental data
