# Supplementary material for: Effectiveness of Medical Music Therapy Practice: Integrative Research Using the Electronic Health Record: Rationale, Design, and Population Characteristics
Source: J Integr Complement Med. 2024 Jan 12;30(1):57–65. doi: 10.1089/jicm.2022.0701 (PMC10795501; doi:10.1089/jicm.2022.0701)
Supplement: Supplemental data [file Supp_DataS1.docx]

**Data collection procedures**

***MT documentation***

During the retrospective study, the music therapy (MT) team primarily used a standardized electronic health record (EHR) documentation template, enabling music therapists to chart all data in one form without having to also spend time documenting in a separate database as in other MT studies.^1,2^ This template enabled structured entry for session-level data including patient-reported outcomes (PROs), goal(s), MT intervention details, and education provided.

***MT referrals***

Patients were eligible to receive MT sessions upon referral from a member of their medical team (e.g., physicians, nurses, social workers, advance practice providers, chaplains, and psychologists). When entering a MT referral, staff would select a reason for referral (e.g., coping, pain management, or anxiety reduction). While the majority of referrals were received electronically via the EHR, some referrals were received verbally or via email. Additionally, some patients received MT services in subsequent hospitalizations following the hospital admission where the initial referral was placed. Thus, not every hospital admission included in this study had an associated EHR MT referral, but every patient seen by MT during the retrospective study was referred to MT either verbally or electronically.

***Data extraction***

Investigators collaborated with a senior developer from the University Hospitals Enterprise Data, Reporting, and Analytics department to extract data from the EHR using structured query language (SQL) procedures. Clinical, demographic, MT documentation, and MT referral data were extracted from the UHCare AllScripts EHR. Financial data including length of stay and primary insurance were extracted from Soarian. Separate extract pairs were executed for each medical center.

***Data cleaning***

Session-level data were manually reviewed to verify conflicts of service (i.e., an attempt was made to see a patient but a session did not occur due to the patient being away from their room, asleep, busy, etc.), flag outpatient sessions and those not written by the UHCWH MT team. Hospital units were categorized by type (e.g., medical/surgical or inpatient oncology) and specialty (e.g., cardiovascular or joint replacement). Regular expressions (i.e., “grepl”) functions in RStudio Version 1.3.1073^3^ were used to extract text matches to specific MT intervention categories (e.g., active music making and live music listening) from free-text fields. MT sessions were then labeled as including an MT intervention or instances of MT education only (i.e., the music therapist assessed the patient and provided education, but did not provide an MT intervention).

The investigators collaborated with the UHCWH MT team to categorize all hospital units where MT sessions occurred by type (e.g., medical/surgical, inpatient oncology, or ICU) and specialty (e.g., cardiovascular, joint replacement, stem cell transplant, or COVID-19). Hospital units were labeled accordingly based on the dates in which specialty care was provided.

***Data combination***

Data types were broken up into patient level, hospital admission level, referral level, and session level. Some patients had multiple hospital admissions, referrals, and MT sessions. For analysis purposes, the final dataset was constructed with repeated measures or one line per MT session.

**References**

1. Lopez G, Christie AJ, Powers-James C, et al. The effects of inpatient music therapy on self-reported symptoms at an academic cancer center: a preliminary report. Support Care Cancer 2019;27(11):4207-4212, doi:10.1007/s00520-019-04713-4

2. Gallagher LM, Lagman R, Rybicki L. Outcomes of Music Therapy Interventions on Symptom Management in Palliative Medicine Patients. Am J Hosp Palliat Care 2018;35(2):250-257, doi:10.1177/1049909117696723

3. R Studio Team. RStudio: Integrated Development for R. RStudio, PBC: Boston, MA; 2020.
